# Tumor Classification Using High-Order Gene Expression Profiles Based on Multilinear ICA

**DOI:** 10.1155/2009/926450

**Published:** 2009-07-20

**Authors:** Ming-gang Du, Shan-Wen Zhang, Hong Wang

**Affiliations:** ^1^School of Urban and Environment Science, Shanxi Normal University, Linfen, Shanxi 041004, China; ^2^Institute of Intelligent Machines, Chinese Academy of Sciences, Hefei, Anhui 230031, China; ^3^College of Mathematics and Computer Science, Shanxi Normal University, Linfen, Shanxi 041004, China

## Abstract

*Motivation*. Independent Components Analysis (ICA) maximizes the statistical independence of the representational components of a training gene expression profiles (GEP) ensemble, but it cannot distinguish relations between the different factors, or different modes, and it is not available to high-order GEP Data Mining. In order to generalize ICA, we introduce Multilinear-ICA and apply it to tumor classification using high order GEP. Firstly, we introduce the basis conceptions and operations of tensor and recommend Support Vector Machine (SVM) classifier and Multilinear-ICA. Secondly, the higher score genes of original high order GEP are selected by using *t-statistics* and tabulate tensors. Thirdly, the tensors are performed by Multilinear-ICA. Finally, the SVM is used to classify the tumor subtypes. *Results*. To show the validity of the proposed method, we apply it to tumor classification using high order GEP. Though we only use three datasets, the experimental results show that the method is effective and feasible. Through this survey, we hope to gain some insight into the problem of high order GEP tumor classification, in aid of further developing more effective tumor classification algorithms.

## 1. Introduction

In the past several years, the DNA microarray technology has attracted tremendous interest in both the scientific community and industry. Generally, developed DNA microarray experiment technology allows the recording of expression levels of thousands of genes simultaneously [1]. Such massive gene expression data gives rise to a number of effective computational challenges. With the wealth of gene expression profiles (GEP), more and more new predictions, clustering, and classifications algorithms have been proposing and used for the GEP analysis [2, 3]. Up to now, many tumor classification methods using GEP are proposed by a number of researchers, and many studies have reported the application of GEP for molecular classification of tumor [4–6]. In GEP data mining, Principal Component Analysis (PCA) is a classic effective tool for analyzing the large-scale GEP [7, 8]. But it ignores all higher-order data relationships—the higher-order statistical dependencies. Independent Components Analysis (ICA) is a useful extension of PCA that has been developed in context with blind separation of independent sources from their linear mixtures [8, 9]. PCA is just sensitive to second-order relationships of the data. However, the ICA model usually leaves some freedom of scaling and sorting by convention, the independent components are generally scaled to unit deviation, while their signs and orders can be chosen arbitrarily. In general, the number of independent components is equal to the number of the observational variables. While the goal of PCA is to minimize the reprojection error from compressed data, while the goal of ICA is to minimize the statistical dependence between the basis vectors. The ICA learns a set of statistical independent components by analyzing the higher-order dependencies in the training samples in addition to the correlations. Such blind separation techniques have been popularly used, for example, in various applications of auditory signal separating, medical signal processing, and so on [10–12]. ICA is capable of extracting biologically relevant gene expression features from microarray data. A number of tumor classification applications for performing ICA have been proposed. Gen et al. (2002) introduced an ICA-based gene classification method. They validated their method by using the yeast GEP during sporulation. Liebermeister [11] applied ICA to microarray data to find independent modes of gene expression. Zhang et al. [13] devised a pattern recognition procedure based on ICA, which is suitable for the identification of diagnostic expression patterns for other human cancers and demonstrates the feasibility of simple and accurate molecular cancer diagnostics for clinical implementation. Zheng et al. [8] performed ICA on the GEP dataset which preprocessed by *t-statistics*, the outputs of ICA were then classified using support vector machine (SVM). Frigyesi et al. [14] applied iterated ICA to three different gene expression datasets to obtain reliable components. They found that many of the low ranking components indeed may show a strong biological coherence and hence be of biological significance. Kong et al. [15] described theoretical frameworks of ICA to further illustrate its feature extraction function in GEP analysis. Biswas et al. [16] applied ICA to gene expression traits derived from a cross between two strains of Saccharomyces cerevisiae and decomposed the data into a set of metatraits, which are linear combinations of all the expression traits.

But, ICA cannot distinguish between high-order GEP that rise from different experiments, or different time, or different studies. These GEP are called high-order GEP. In practice, the structure of GEP integrated from different studies is of an order higher than that of a matrix. These datasets can be tabulated a tensor. If we deal with these GEP respectively or unfold the tensor into a matrix, these degrees of freedom are lost and much of the information in the data tensor might also be lost. This problem is addressed by multilinear framework. Whereas ICA employs linear (matrix) algebra, Multilinear ICA model exploits tensor algebra [17, 18]. Multilinear ICA is able to learn the interactions of multiple samples (genes) inherent to high-order dataset formation and separately encode the higher-order statistics of each of these factors. It has been used widely in image recognition [17–20]. Omberg et al. [21] described a multilinear high-order SVD, reformulated to decompose a data tensor into a linear superposition of rank-1 subtensors, and provided an integrative framework for high-order GEP analysis from different studies, where significant subtensors represent independent biological programs or experimental phenomena. A quick survey of biological literatures shows that multilinear ICA is still seldom used in bioinformatics. In this paper, we apply Multilinear ICA to tumor classification using high-order GEP.

This paper is organized as follows. Section 2 briefly discusses some mathematical backgrounds, including tensor, multilinear ICA model, and SVM classifier and introduces the gene selection strategy based on the *t-statistics * and Multilinear ICA model of high-order GEP dataset. In Section 3, a classification method using multilinear ICA is proposed, and the predication results for applying the method to the high-order GEP are given. Some conclusive remarks and future works are included in Section 4.

## 2. Methods

In recent years the tensor analysis in pattern recognition and other areas has attracted more and more attention. Tensor means multidimensional or multimode array. Often the data have a multidimensional structure and it is then somewhat unnatural to organize them as matrices or vectors. As a simple example, each GEP is a two-dimensional data array, that is, a matrix. Then many GEP from different studies are 3-dimensional data array, which can be easy expressed by a third-order tensor. Though tensor analysis has been used for a long time in many areas, it is seldom used in GEP analysis. So it is necessary to introduce the basis conceptions and operations of tensor [22, 23].

### 2.1. Mathematical Background of Tensor

**Figure 1:**
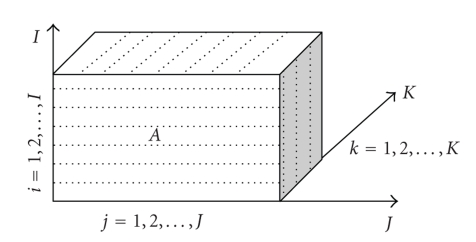
Third-order tensor.

A tensor is a multidimensional array. Roughly speaking, a scalar is a 0-order tensor, an *n*-vector is a 1-order tensor of size *n*, and an *m* × *n* matrix is a 2-order tensor of size *m* × *n*. An *N*th*- *order tensor, denoted as*A* = {*a*_*i*_1_*i*_2_⋯*i*_*N*__} ∈ ℝ^*I*_1_×*I*_2_×⋯×*I*_*N*_^, is a generalization of these algebraic objects to one with *N* indices, where *a*_*i*_1_*i*_2_⋯*i*_*N*__ denotes its random element. The dimension of *A* along the different orders is given by *I*_*i*_ (*i* = 1, 2,…, *N*). Tensors are often found in differential geometry where they most of the time (if not exclusively) represent multilinear operators. A third-order tensor, denoted as *A* = {*a*_*ijk*_} ∈ ℝ^*I*×*J*×*K*^, has three indices as shown in Figure 1.

The starting point of the derivation of a multilinear SVD will be to consider an appropriate generalization of the link between the column (row) vectors and the left (right) singular vectors of a matrix. To be able to formalize this idea, we introduce “tensor unfolding.” There are several ways to do so. To avoid confusion, we will stick to one particular ordering of the column (row,…) vectors. One particular type of “tensor unfolding” will prove to be particularly useful, namely, the matrix representation of a given tensor in which all its column (row,…) vectors are simply stacked one after another. Simply, for a 3-order tensor *A* ∈ ℝ^*n*×*n*×*n*^, these unfolding procedures can be visualized, *A*_(*i*)_ ∈ ℝ^*n*×*n*^2^^ (*i* = 1, 2, 3) is expressed in detail as follows:

(1) $$\begin{array}{ll} A_{\left(1\right)} & =\left(\begin{array}{cccc} A\left(:,1,:\right) & A\left(:,2,:\right) & \cdots & A\left(:,n,:\right) \end{array}\right),\\ A_{\left(2\right)} & =\left(\begin{array}{cccc} A\left(:,:,1\right) & A\left(:,:,2\right) & \cdots & A\left(:,:,n\right) \end{array}\right),\\ A_{\left(3\right)} & =\left(\begin{array}{cccc} A\left(1,:,:\right) & A\left(2,:,:\right) & \cdots & A\left(n,:,:\right) \end{array}\right). \end{array}$$

For two tensors *A*, *B* ∈ ℝ^*I*_1_×*I*_2_×⋯×*I*_*N*_^, their inner product, denoted as 〈*A*, *B*〉, is defined in a straightforward way as

(2) $$\langle A,B\rangle =\overset{I_{1}}{\underset{i_{1}=1}{\sum }}\;\overset{I_{2}}{\underset{i_{2}=1}{\sum }}\cdots \overset{I_{N}}{\underset{i_{N}=1}{\sum }}a_{i_{1},i_{2},\ldots ,i_{N}}\cdot b_{i_{1},i_{2},\ldots ,i_{N}}.$$

The norm of a tensor *A* ∈ ℝ^*I*_1_×*I*_2_×⋯×*I*_*N*_^ is defined as

(3) $$\left\|A\right\|=\sqrt{\langle A,A\rangle }.$$

We regard that two tensors are called orthogonal when their inner product is 0. The tensor distance between *A* and *B* is expressed as follows:

(4) D(A,B)=‖A−B‖.

### 2.2. Mathematical Background of Multilinear ICA

Independent component analysis (ICA) is a valid data analysis technique for uncovering independent components which underlie the observational data (Lieven et al., 2000). This technique seeks the linear transformation of the original data to have a mutually independent representation. ICA is a linear analysis method, which can remove all linear correlations. But, it is not well suited to the representation of high-order GEP ensembles. To remedy this shortcoming, we introduce the Multilinear ICA as follows [19, 24, 25].

Recall the classical SVD of a matrix,

(5a) A=UΣV.

Since *A* and Σ are matrices, they are also regarded as 2-order tensors. It is not hard to understand and verify following representation by tensor product. We can express the SVD in terms of the *n*-mode product,

(5b) $$A=\Sigma \times_{1}U\times_{2}V.$$

Naturally for a general tensor *A* ∈ *R* ^*I*_1_×*I*_2_×⋯×*I*_*N*_^, high-order SVD [26, 27] is obtained by decomposing the tensor *A* as the tensor product of an*N- *order tensor *S * and a series of matrices *U*_*n*_ (*n* = 1, 2,…, *N*), written as follows:

(6) $$A=S\times_{1}U_{1}\times_{2}U_{2}\times_{3}\cdots \times_{N}U_{N}.$$

Where *S* is called the core tensor, *U*_*n*_ (*n* = 1, 2,…, *N*) is a mode matrix spanning the column space of *A*_(*n*)_, which is the mode-*n* flattening of *A*.

The core tensor *S* is analogous to the diagonal singular value matrix in conventional matrix SVD (although it does not have a simple, diagonal structure). The core tensor governs the interaction between the mode matrices *U*_1_, *U*_2_,…, *U*_*N*_, which contain the orthonormal vectors spanning the column space of matrix *A*_(*n*)_ resulting from the *n*th-mode flattening of tensor *A*.

For third-order tensor *A* ∈ ℝ^*I*×*J*×*K*^, *A* can be written as the product

(7) $$A=S\times_{1}U_{1}\times_{2}U_{2}\times_{3}U_{3},$$

with the following properties.

- *U*_1_ ∈ ℝ^*I*×*I*^, *U*_2_ ∈ ℝ^*J*×*J*^, and *U*_3_ ∈ ℝ^*K*×*K*^ are orthogonal matrices.
- *S* is a real tensor of the same dimensions as *A* and is all orthogonal, that is, slices along any mode are orthogonal, let *i* ≠ *j*, 〈*S*(*i*, :, :), *S*(*j*, :, :)〉 = 〈*S*(:, *i*, :), *S*(:, *j*, :)〉 = 〈*S*(:, :, *i*), *S*(:, :, *j*)〉 = 0.
- The *i*-mode singular values are the diagonal elements of Σ^(*i*)^ = diag (*σ*_1_^(*i*)^, *σ*_2_^(*i*)^,…, *σ*_*n*_^(*i*)^). The norms of the slices along every mode are ordered, *σ*_1_^(*i*)^ ≥ *σ*_1_^(*i*)^ ≥ *σ*_2_^(*i*)^ ≥ ⋯ ≥ *σ*_*n*_^(*i*)^ ≥ 0, *i* = 1, 2, 3.

For 1-mode singular values of the matricized tensor *A*_(1)_, we have *σ*_*j*_^(1)^ = ‖*S*(*j*, :, :)‖, *j* = 1, 2,…, *n*, and *σ*_1_^(1)^ ≥ *σ*_2_^(1)^ ≥ ⋯ ≥ *σ*_*n*_^(1)^ ≥ 0. The ordering property implies that, loosely speaking, the “energy” or “mass” of the core tensor *S* is concentrated in the vicinity of the point (1, 1, 1) nearby. This property makes it possible to use the high-order SVD for data compression.

In fact, we can compute the Multilinear ICA by the following two steps.

1. For each *i* = 1, 2, 3, compute *U*_*i*_ by computing SVD of *A*_(*i*)_ = *U*_*i*_Σ*V* ^*T*^.
2. Solve for the core tensor as

(8) $$S=A\times_{1}U_{1}^{-T}\times_{2}U_{2}^{-T}\times_{3}U_{3}^{-T}.$$

In ICA, there is a strategy for multilinear ICA. The architecture results in a factorial code, where each set of coefficients that encodes samples of tumor, genes, spanning data sources, and so forth is statistically independent. Flattening the data tensor *A* in the *n*th mode and computing the ICA, we obtain

(9) $$\begin{array}{ll} A_{\left(n\right)}^{T} & =U_{n}\Sigma_{n}^{T}V_{n}^{T}\\ & =\left(U_{n}W_{n}^{-1}\right)\left(W_{n}\Sigma_{n}^{T}V_{n}^{T}\right)\\ & =C_{n}K_{n},\;n=1,2,3, \end{array}$$

where the mode matrices are given by *C*_*n*_ = *U*_*n*_ *W*_*n*_^−1^.

The architecture results in a set of basis vectors which are statistically independent across the different modes. We can derive the relationship between *N- *mode ICA and *N*-mode SVD in the context of the architecture as follows:

(10) $$\begin{array}{ll} A & =Z\times_{1}U_{1}\times_{2}U_{2}\times_{3}U_{3}\\ & =Z\times_{1}U_{1}W_{1}^{-1}W_{1}\times_{2}U_{2}W_{2}^{-1}W_{2}\times_{3}U_{3}W_{3}^{-1}W_{3}\\ & =Z\times_{1}\left(U_{1}W_{1}^{-1}\right)W_{1}\times_{2}\left(U_{2}W_{2}^{-1}\right)W_{2}\times_{3}\left(U_{3}W_{3}^{-1}\right)W_{3}\\ & =Z\times_{1}C_{1}W_{1}\times_{2}C_{2}W_{2}\times_{3}C_{3}W_{3}\\ & =\left(Z\times_{1}W_{1}\times_{2}W_{2}\times_{3}W_{3}\right)\times_{1}C_{1}\times_{2}C_{2}\times_{3}C_{3}\\ & =S\times_{1}C_{1}\times_{2}C_{2}\times_{3}C_{3}, \end{array}$$

where the core tensor is *S* = *Z* × _1_ *W*_1_ × _2_ *W*_2_ × _3_ *W*_3_.

### 2.3. Mathematical Background of Gene Selection Strategy

Among a large number of genes of GEP, only a small part may benefit the correct classification of tumor subtypes. The large rest of genes has little impact on the classification. Even worse, some genes may act as “noise” and depress the classification accuracy. To obtain higher classification accuracy, we need to pick out a gene subset which benefits the tumor classification most.

*T*-statistics is a statistical method. It is applied to measuring how large the difference is between the distributions of two groups of the samples. For a single gene, if it shows larger distinction between two groups, it is more important for the classification of the two groups. To find the genes that contribute most to the classification,* t-statistics * has been used in gene selection in recent years [8, 28].

Selecting important genes using *t-statistics * involves three steps. Firstly, a score based on *t-statistics * (named *S*-score) is calculated for each gene by the following (11):

(11) $$s\left(g_{j}\right)=\left\|\frac{\mu_{j}^{1}-\mu_{j}^{2}}{\sigma_{j}^{1}+\sigma_{j}^{2}}\right\|.$$

This step allows to find the important genes that help to discriminate between two classes by calculating a score for each gene *g*_*j*_ based on the mean *μ*_*j*_^1^ (resp., *μ*_*j*_^2^) and the standard deviation *σ*_*j*_^1^ (resp., *σ*_*j*_^2^) of each class of the samples.

Secondly, all the genes are rearranged according to their *t*-score. The gene with the largest *t*-score is put in the first place of the ranking list, followed by the gene with the second largest *t*-score, and so on.

Finally, only some top genes in the list are used for classification. We select a set of genes corresponding to the top ranked to be used as initial informative genes. The standard *t-statistics * is only applicable to measure the difference between two groups. Therefore, when the number of classes is more than two, we need to modify the standard *t-statistics*.

### 2.4. Mathematical Background of SVM Classifier

Support vector machine (SVM) is an area of statistical learning, subject to extensive research [29]. The SVM is based on the principle of risk minimization and thus provides good generalization control. This allows one to work with datasets that contain many irrelevant and noisy features. Using nonlinear kernels, SVM can model nonlinear dependences among features and the target, which may prove advantageous for the classification problems. When SVM is used for tumor gene classification, it can separate a given set of binary labeled training data with a hyperplane that is maximally distant from them (the maximal margin hyperplane) [8, 30].

Because there are only few samples of the GEP achieved in general, we use SVM [5] as the classifier in our feature selection study, which have been proven to be very useful for classifying the gene expression data.

A MATLAB toolbox implementing SVM is freely available for academic purposes, and we can download from: http://www.isis.ecs.soton.ac.uk/resources/svminfo/.

### 2.5. Multilinear-ICA Model of High Order GEP Dataset

The structure of GEP integrated from different studies or experiments is of an order higher than that of a matrix, we generally call it spanning datasets. Now let the tensor *A* = {*a*_*ijk*_} ∈ ℝ^*I*×*J*×*K*^ denote the GEP of spanning dataset, of size *I*-sample × *J*-gene × *K*-dataset, and *a*_*ijk*_ is the expression level of the *j*th gene in the *i*th sample of *k*th dataset, in general *I* ≪ *J*, *K* ≪ *I*. Each column vector of tensor *A*, that is *A*_:*jk*_, lists the GEP measured under the *j*th gene and *k*th dataset. The row vectors, *A*_*i*:*k*_ and *A*_*ij*:_, list the GEP measured for the *i*th sample under the *k*th dataset across all genes, and under the *j*th gene across all datasets, respectively. We suppose that all data have already been preprocessed and normalized, that is, every gene of GEP has mean zero and standard deviation 1.

The following discuss computational methods for the best multilinear rank approximation problem:

(12) $$\underset{B}{\min\;}\;\left\|A-B\right\|,$$

where *A* is a given GEP-tensor and *B* is unknown GEP-tensor.

Our goal is to seek the best low order multilinear dimension approximation tensor *B*. This is a generalization of the best low dimension tensor approximation problem. It is well known that for matrix the solution is given by truncating the singular values in the SVD of the matrix. But for tensor in general, the truncated tensor-SVD does not give an optimal approximation.

A third-order GEP-tensor *B* ∈ ℝ^*I*×*J*×*K*^ with rank (*γ*_1_, *γ*_2_, *γ*_3_) can be written as the product

(13) $$B=S\times_{1}U_{1}\times_{2}U_{2}\times_{3}U_{3},$$

where *S* ∈ ℝ^*γ*_1_×*γ*_2_×*γ*_3_^ is a tensor, and *U*_(1)_ ∈ ℝ^*I*×*γ*_1_^, *U*_(2)_ ∈ ℝ^*J*×*γ*_2_^, and *U*_(3)_ ∈ ℝ^*K*×*γ*_3_^ are matrices with orthonormal columns. The approximation problem is equivalent to a nonlinear optimization problem defined on a product of Grassmann manifolds.

We want to find a tensor *B* of the form *B* = *λU* ^(1)^ ∘ *U* ^(2)^ ∘ *U* ^(3)^. It fixes all *U*-vectors except *U* ^(1)^ and then solves for the optimal *U* ^(1)^, likewise for *U* ^(2)^, *U* ^(3)^, cycling through the indices until the specified number of iterations is exhausted. These steps [31] are explained as follows:

*In *: GEP-tensor *A* = {*a*_*ijk*_} ∈ ℝ^*I*×*J*×*K*^.

*Out *: GEP-tensor *B* ∈ ℝ^*I*×*J*×*K*^, an estimate of the best rank-1 approximation of *A*.

1. Compute initial values. Let *U*_0_^(*i*)^ be the dominant left singular vector of *A*_(*i*)_, *i* = 1, 2, 3.
2. For *k* = 0,1, 2,… (until converged), do what follows.
  (14) $$\begin{array}{cc} \text{For}\; & i=1,2,3,\\ & {\tilde{U}}_{k+1}^{\left(i\right)}=A\times_{n}{\left\{U_{k}\right\}}^{-1},\\ & \sigma_{k+1}^{\left(i\right)}=\left\|{\tilde{U}}_{k+1}^{\left(i\right)}\right\|,\\ & U_{k+1}^{\left(i\right)}=\frac{{\tilde{U}}_{k+1}^{\left(i\right)}}{\sigma_{k+1}^{\left(i\right)}},\\ \text{end} & \end{array}$$
3. Let *σ* = *σ*_*K*_, and let {*U*} = {*U*_*K*_}, where *K* is the index of the final result of step 2.
4. Set *B* = *σU* ^(1)^ ∘ *U* ^(2)^ ∘ *U* ^(3)^.

This algorithm is important in practical applications, for the different rank-1 terms can often be related to different “mechanisms” that have contributed to the higher-order tensor, in addition, sufficiently mild uniqueness conditions enable the actual computation of these components (without imposing orthogonality constraints, as in the matrix case).

In general, the number of genes in a single sample is in the thousands. So the above procedure can be used to compress the High-order GEP.

### 2.6. Tumor Classification Method

To simplify the computation, we normalized the expression values for each of the genes such that each sample has zero mean and unit variance. We chose respectively larger-score genes from all GEP datasets using the method described in Section 2.3. We divide each dataset into two parts, training subdataset and testing subdataset, and tabulate two tensors, training tensor *A*_tn_ and testing tensor *A*_tt_, respectively. We performed Multilinear ICA on training tensor *A*_tn_ to produce a core tensor *S*_tn_ and three matrixes *U*_1_, *U*_2_, and *U*_3_ such that

(15) $$A_{\text{tn}}=S_{\text{tn}}\times_{1}U_{1}\times_{2}U_{2}\times_{3}U_{3}.$$

Here, the core tensor *S*_tn_ contains the coefficients (representations) of the multilinear combination of statistically independent sources (rows of *U*_*i*_, *i* = 1, 2, 3) that comprise *A*_tn_. From the testing tensor *A*_tt_ and *U*_1_, *U*_2_, *U*_3_, we can achieve core tensor *S*_tt_ by the following equation:

(16) $$S_{\text{tt}}=A_{\text{tt}}\times_{1}U_{1}^{-T}\times_{2}U_{2}^{-T}\times_{3}U_{3}^{-T}.$$

After achieving the representations of the training and test data using *t-statistics * and Multilinear ICA, the final step is to classify the dataset. We unfold the tensors *S*_tn_ and *S*_tt_, obtain two matrices (*S*_tn_)_(1)_ and (*S*_tt_)_(1)_, and truncate them. And then, we use (*S*_tn_)_(1)_ and its corresponding label to train SVM classifier. Finally, we import (*S*_tt_)_(1)_ to SVM and export its corresponding label to assess the performance.

## 3. Results

To verify the classification abilities of the proposed algorithm, the experimental results are presented in this section. *t-statistics* is first used to select gene which with high score, multilinear ICA model is acted on the chosen training GEP-tensor to extract independent eigenarrays, and then SVM is applied to classify the tumor samples using their representations corresponding to independent eigenarrays.

### 3.1. Datasets

There are three available GEP datasets, leukemia tumor and lung tumor. Two publicly leukemia datasets are downloaded from the web sites, http://www.broad.mit.edu/cgi-bin/cancer/datasets.cgi and http://www.genome.wi.mit.edu/MPR. The lung tumor dataset can be downloaded from the web site http://www.broad.mit.edu/cgi-bin/cancer/datasets.cgi The descriptions of three datasets are shown in Table 1.

Because we have not obtained more available High-order GEP from public web set, in order to validate the proposed algorithm, we have to divide a large tumor dataset to *n* small parts, which regard as *n* datasets obtained from *n* different experiments or *n* different studies.

**Figure 2:**
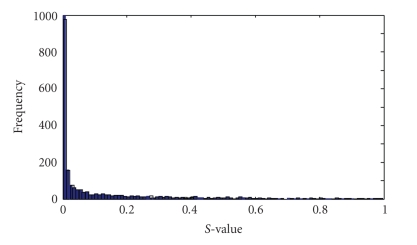
The gene distribution frequency versus gene *S*-values.

All the datasets are normalized so that they have zero means and standard deviations. After being normalized, the genes in the GEP are ranked by *t-statistics*. The *S*-value distribution of every gene is shown in Figure 2 on leukemia dataset 1.

From Figure 2, we can see that the number of genes with very little *S*-value is very large. That is to say, the vast majority of genes have little or not contribution to tumor classification. In general, we have reason to simply select 200 top ranked genes from the three datasets for Multilinear ICA, respectively.

### 3.2. Two-Fold Crossvalidated on Leukemia Datasets

In order to buildup a tensor of spanning datasets, we chose randomly 38 samples from dataset 2 as many as all from the dataset 1. We design an experiment on all 38 × 2 samples using 2-fold crossvalidated to evaluate the classification model. In these datasets, we chose 200 larger-score genes from two datasets using the *t-statistics * described in Section 2.3 for analyses and constitute training tensor and testing tensor, and all data samples are already assigned to a training set *A*_tn_ or testing set *A*_tt_, as shown in Table 2.

**Figure 3:**
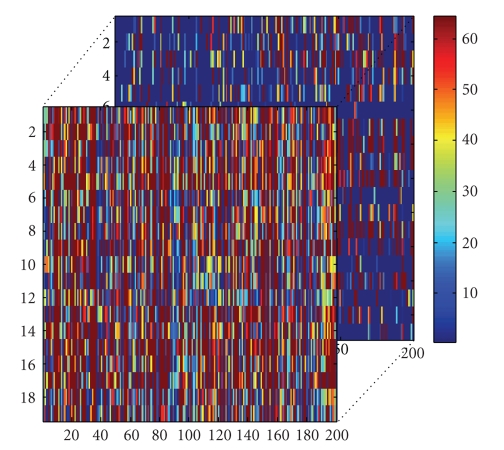
Training tensor *A*_tn_.

**Figure 4:**
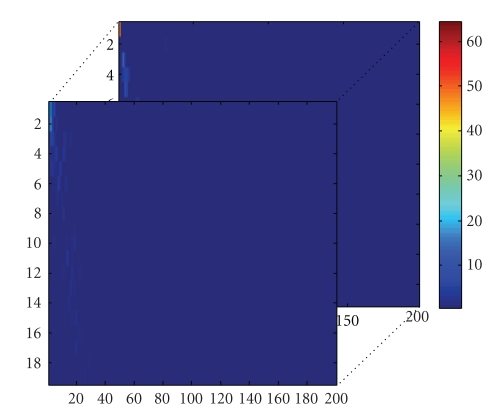
Training core tensor *S*_tn_.

By above analysis, the original training set *A*_tn_ and testing set *A*_tt_ are all 200 × 19 × 2 tensors. We performed Multilinear ICA on training tensor *A*_tn_ to obtain a core tensor *S*_tn_ and three matrixes *U*_1_, *U*_2_, and *U*_3_; they are as shown in Figures 3-4. From Figure 4, we find that many elements of *S*_tn_ are very small or zero.

From the testing tensor *A*_tt_ and three matrixes *U*_1_, *U*_2_, and *U*_3_, we can obtain the testing core tensor *S*_tt_ by (16). Then we unfold the tensors *S*_tn_ and *S*_tt_, obtaining two matrices (*S*_tn_)_(1)_ and (*S*_tt_)_(1)_. We then use (*S*_tn_)_(1)_ and their corresponding label to train the SVM classifier with Gaussian kernels and finally use (*S*_tt_)_(1)_ and their corresponding label to assess the performance. The statistical mean correct classification result is 99%.

### 3.3. LOO-CV on Two Leukemia Datasets

Because of a fat lot datasets at hand, we do the same experiment by leave-one-out cross validated (LOO-CV), that is, the training set *A*_tn_ is a tensor 200 × 37 × 2, and the testing set *A*_tt_ is a tensor 200 × 1 × 2. The classification process is in principle similar to the one described above. The statistical mean correct classification result is 99.80%.

### 3.4. LOO-CV on “Three” Leukemia Datasets

**Figure 6:**
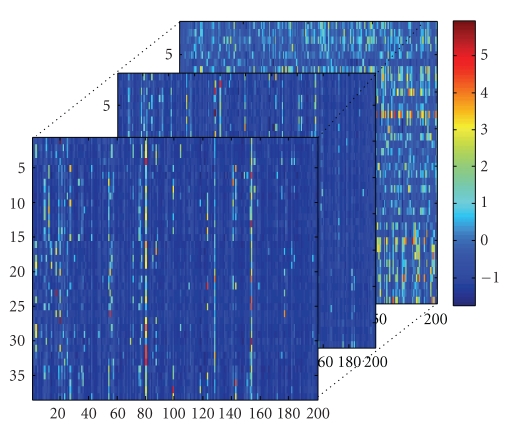
Training tensor *A*_tn_.

**Figure 7:**
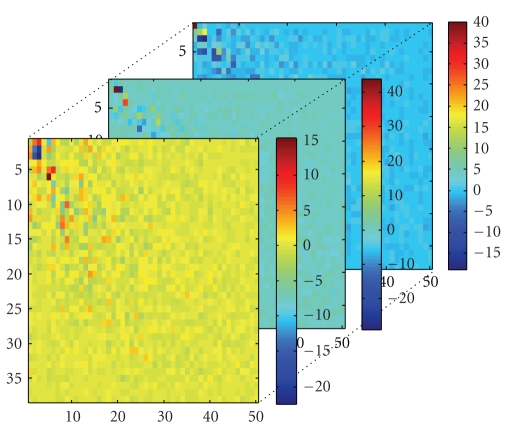
Training core tensor *S*_tn_.

**Figure 8:**
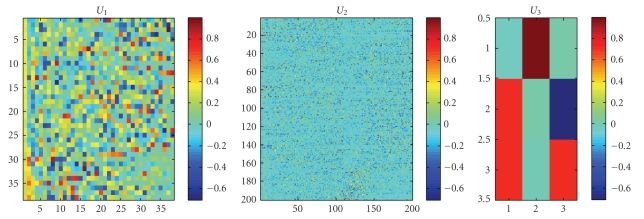
Three matrixes *U*_1_, *U*_2_, and *U*_3_.

We divide the above dataset 2 into two parts, each part has 38 samples. Note that there are 4 samples in two parts synchronously. Now we have three datasets and assign them to a training set or testing set. We design that the training set *A*_*tn*_ is tensor 200 × 37 × 3, and the testing set *A*_tt_ is 200 × 1 × 3. We performed Multilinear ICA on training tensor *A*_tn_. We can obtain a core tensor (*S*_tn_)_(1)_ and three matrixes *U*_1_, *U*_2_, and *U*_3_, as shown in Figures 6, 7, and 8. Then *S*_tt_ can be obtained from (16). After unfolding *S*_tn_ and *S*_tt_ as matrixes(*S*_tn_)_(1)_ and (*S*_tt_)_(1)_, respectively, and achieving the representations of the training and test data, we then use (*S*_tn_)_(1)_ and their corresponding label to train SVM, and finally use (*S*_tt_)_(1)_ and their corresponding label to assess the performance. The classifying process is in principle similar to the ones described above. The statistical mean correct classification result is 99.54%. We find that this result is little smaller than the result in Section 3.3. The reason is that the Leukemia Dataset 2 is divided into two parts.

From the above experimental results, we can see that with the gene of spanning datasets decrease used in Multilinear ICA, the classification accuracy of the spanning Leukemia datasets is high, which means that the result is effective.

### 3.5. LOO-CV on “Three”-Order Lung Datasets

**Figure 9:**
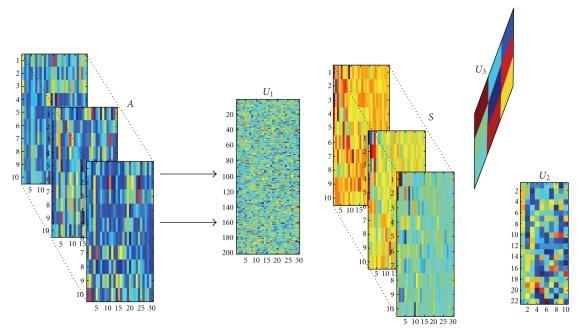
MICA for three-order lung microarray.

Similar to Section 3.4, we firstly chose 200 genes using the *t-statistics * described in Section 2.3 for analyses, then select dividing 180 samples of the lung GEP dataset 3 into three parts as training set, each part having 60 samples, while a rest sample as test set. We design that the training set *A*_tn_ is tensor 200 × 60 × 3, and the testing set *A*_tt_ is 200 × 1 × 3. After performing Multilinear ICA on training tensor *A*_tn_, a core tensor *S*_tn_ and three matrixes *U*_1_, *U*_2_, and *U*_3_ are obtained, as shown in Figure 9, then *S*_tt_ can be obtained from (16).

After unfolding *S*_tn_ and *S*_tt_ as matrixes(*S*_tn_)_(1)_ and (*S*_tt_)_(1)_, respectively, and achieving the representations of the training and test data, we then use (*S*_tn_)_(1)_ and their corresponding label to train SVM, and finally use (*S*_tt_)_(1)_ and their corresponding label to assess the performance. The statistical mean correct classification result is 90.26%.

To show the efficiency and the feasibility of the proposed method, we compare our method with other two methods, SVM and ICA + SVM [8]. The classification results are listed in Table 3 for comparison.

From Table 3, it is found that the result of ICA + SVM is little better than the proposed method on the lung datasets. The reason is also that the complete lung data is divided into three parts.

The experiment results demonstrate that our method achieves better classification rate. When the microarray data is high-order integrated from different studies, if we unfold the data into matrix, the structure of the data is break and most of the information in the tensor data might be lost. The proposed method can analyze and dispose synchronously the high-order GEP datasets. We could experience the superiority by using our proposed method on high-order data.

## 4. Conclusions

The tumor classification based on GEP is a challenging task in bioinformatics. The developed DNA microarray experiment technology has resulted in expression levels of thousands of genes being recorded over just a lot of different samples. ICA is a novel tool on the single GEP. But it is not available for the high-order datasets integrated from different studies or different experimental setting. Considering the biological significance, we think that the classification using a relatively large number of genes of spanning datasets may be more reasonable. For this reason, a new classification scheme for High-order GEP is proposed. The method involves dimension reduction of high-order High-order GEP using Multilinear ICA, followed by using *t-statistics* and the classification applying SVM. The experimental results show that our proposed method is effective. The method only provides an integrative framework for higher-order tumor classification using High-order GEP. However, there is still a great amount of work that needs to be done in order to achieve the goal of tumor classification of spanning datasets. Further work needs doing to apply our methods to other high order GEP based on hard classified tumors.

## Figures and Tables

**Figure 5:**
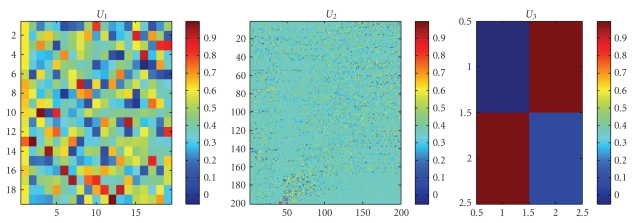
Three matrixes *U*_1_, *U*_2_, and *U*_3_.

**Table 1:** Descriptions of three original tumor datasets.

Tumor dataset	#Gene	#Sample	Subtype 1	Subtype 2
Leukemia dataset 1	7,129	38	27(ALL)	11(AML)
Leukemia dataset 2	7,129	72	47(ALL)	25(AML)
Lung dataset 3	12,533	181	32	149

**Table 2:** Distribution of two tumor datasets in our experiments.

Tumor dataset	Choosing gene	Choosing sample	Training sample	Testing sample
Leukemia dataset 1	200	38	19	19
Leukemia dataset 2	200	38	19	19

**Table 3:** Classification results on three tumor datasets by LOO-CV.

	Dataset			
Method	Leukemia dataset 1	Leukemia dataset 2	Leukemia dataset 1 + dataset 2	Lung dataset 3
SVM	95.76	94.32	90.75	88.69
ICA + SVM	99.15	99.45	95.48	90.44
Multilinear ICA + SVM	—	—	99.80	90.26
			99.54	
